# Electrocardiographic substrates of arrhythmias in patients with end-stage and chronic kidney diseases: a case–control study

**DOI:** 10.1186/s43044-023-00338-5

**Published:** 2023-02-21

**Authors:** Hesham Yehia, Ghada Youssef, Mona Gamil, Mahmoud Elsaeed, Khaled M. Sadek

**Affiliations:** 1 Cairo University, Cairo, Egypt; 2 Cardiovascular Department, Kasr Al Ainy School of Medicine, Cairo University, Cairo, Egypt; 3 Internal Medicine Department, Kasr Al Ainy School of Medicine, Cairo University, Cairo, Egypt

**Keywords:** Chronic kidney disease, End-stage renal disease, Arrythmia, Electrocardiogram

## Abstract

**Background:**

Cardiovascular disease (CVD) is the most common cause of death in patients with renal diseases. Cardiac arrhythmia and sudden cardiac death are particularly important, and the burden is higher in patients on hemodialysis. The aim of this study is to compare specific ECG changes as markers of arrhythmias in patients with CKD and patients with end-stage renal disease (ESRD); all without clinically manifest heart disease, with normal control subjects.

**Results:**

Seventy-five ESRD patients on regular hemodialysis, 75 patients with stage 3–5 CKD and 40 healthy control subjects were included. All candidates were subjected to thorough clinical evaluation and laboratory tests including serum creatinine, glomerular filtration rate calculation, serum potassium, magnesium, calcium, phosphorus, iron, parathyroid hormone, and total iron binding capacity (TIBC). Resting twelve-lead ECG was done to calculate P wave dispersion (P-WD), corrected QT interval, QTc dispersion, **T**peak-**T**end interval (**T**p-e), and Tp-e/QT. Patients with ESRD had a significantly higher QTc dispersion (*p* < 0.001) and P-WD (*p* = 0.001) when compared to the other 2 groups. In the ESRD group, males had a significantly higher P-WD (*p* = 0.045), insignificantly higher QTc dispersion (*p* = 0.445), and insignificantly lower Tp-e/QT ratio (*p* = 0.252) as compared to females. Multivariate linear regression analysis for ESRD patients showed that serum creatinine (*β* = 0.279, *p* = 0.012) and transferrin saturation (*β* =  − 0.333, *p* = 0.003) were independent predictors of increased QTc dispersion while ejection fraction (*β* = 0.320, *p* = 0.002), hypertension (*β* =  − 0.319, *p* = 0.002), hemoglobin level (*β* =  − 0.345, *p* = 0.001), male gender (*β* =  − 0.274, *p* = 0.009) and TIBC (*β* =  − 0.220, *p* = 0.030) were independent predictors of increased P wave dispersion. In the CKD group, TIBC (*β* =  − 0.285, *p* = 0.013) was an independent predictor of QTc dispersion while serum calcium (*β* = 0.320, *p* = 0.002) and male gender (*β* =  − 0.274, *p* = 0.009) were independent predictors of Tp-e/QT ratio.

**Conclusions:**

Patients with stage 3–5 CKD and those with ESRD on regular hemodialysis exhibit significant ECG changes that are considered substrates for ventricular as well as supraventricular arrhythmias. Those changes were more evident in patients on hemodialysis.

## Background

Chronic kidney disease (CKD) is a serious worldwide health problem. Cardiovascular disease (CVD) is the commonest cause of death in CKD as it accounts for 43% of all-cause mortality in hemodialysis patients [1]. Arrhythmia is common in patients with CKD and sudden cardiac death accounts for 25% of deaths in dialysis patients [2].

Several electrocardiographic (ECG) methods can be used to assess the risk of arrhythmia including measurement of the P wave dispersion (P-WD), corrected QT interval (QTc), peak of T wave to end of T wave interval (Tp-e), and QTc dispersion on the standard 12-lead ECG. Limited studies have assessed the resting 12-lead ECG as a screening tool in intermediate risk populations [3].

This study aimed to evaluate P-WD, QTc interval, QTc dispersion, Tp-e and Tp-e/QT ratio in patients with stage 3–5 CKD not on renal replacement therapy (RRT) and end-stage renal disease (ESRD) patients on regular hemodialysis; all without clinically manifest heart disease; compared to values in normal control subjects.

## Methods

Seventy-five ESRD patients on regular hemodialysis, 75 stage 3–5 CKD patients (eGFR < 60 mL/min) without RRT and 40 healthy control subjects were included.

Patients excluded from the study were those on drugs that were known to prolong the QT interval (e.g., antibiotics as clarithromycin, antifungal as fluconazole, antiarrhythmics as amiodarone, antipsychotic as chlorpromazine), patients with structural heart diseases (e.g., significant valvular lesions), patients with impaired left ventricular systolic function (defined as ejection fraction < 50%) and patients with atrial fibrillation (because of the marked variabilities in the ECG intervals).

Patients were chosen from the Internal Medicine inpatient department and outpatient clinic and the chronic hemodialysis unit at Cairo University Hospital. The protocol of the study was approved by the ethics committee of the Faculty of Medicine, Cairo University, Cairo, Egypt.

After obtaining written, informed consents, individuals were subjected to thorough clinical evaluation (age, sex, history of hypertension (HTN) or diabetes mellitus (DM)) and laboratory tests including complete blood count, urea, creatinine, glomerular filtration rate (eGFR) (calculated by CKD-EPI equation) [4], serum potassium, magnesium, calcium, phosphorus, parathyroid hormone (PTH), iron and total iron binding capacity (TIBC).

Twelve-lead ECG was performed by General Electric Prucka (GE Healthcare Technologies, WI, USA) electrophysiological study machine. The machine was set to draw cardiac electric waves at a standard voltage (10 mm) and speed (25 mm/s). Corrected QT (QTc), QTc dispersion, P-WD, Tp-e interval and Tp-e/QT ratio were measured as follows:

- Corrected QT interval:
  - The QT interval was measured in each of the 12 leads, from QRS complex onset to the end of the T wave off-set, as defined by the return of the terminal T wave (upright or inverted) to isoelectric TP baseline.
  - The QT interval was then corrected for the heart rate (QTc) using Bazett's formula:$QTc=\frac{QT}{\sqrt{RR}}$ [5]. The maximum and the minimum QTc values were reported.
  - The average of the 12-lead QTc intervals was calculated. Prolonged QTc was diagnosed when QTc > 450 ms in males and > 470 ms in females [6].
- QTc dispersion was calculated as the difference between the maximum and the minimum QTc. QTc dispersion should be between 30 and 60 ms in normal subjects [7].
- P-WD was calculated as the difference between the maximum and the minimum P wave durations [8]. The normal value of P-WD is 29 ± 9 ms. A maximum P-WD value is 36 ms [9].
- T wave peak to end interval
  - Tp-e interval was measured in each of the 12 leads, as the distance from the peak to the end of the T wave.
  - The mean of the Tp-e intervals was then calculated.
- The ratio between the mean of the Tp-e intervals and the mean of the QTc intervals was calculated [10]. The normal Tp-e/QT ratio should be ≤ 0.25 [11].

### Statistical methods

The collected data were revised, coded, tabulated and introduced to a PC using statistical package for social science (SPSS 15.0.1 for windows; SPSS Inc, Chicago, IL, 2001). Data were presented as mean and standard deviation for quantitative parametric variables. Frequency and percentages were used to describe qualitative data. Student *t*-test, Mann Whitney and ANOVA tests were used to compare means of quantitative data while Chi square/Fisher exact test was used to compare qualitative data. Pearson correlation was used to correlate quantitative data. Regression analysis was used to detect independent predictors of ECG changes. *P*-value < 0.05 was considered statistically significant.

## Results

Table 1 shows the basic clinical and laboratory findings in the 3 study groups. Subjects in the control group were younger, less anemic and had a lower prevalence of hypertension.

**Table 1:** Clinical and laboratory data of the study participants

	ESRD (*n* = 75) Mean ± SD	CKD (*n* = 75) Mean ± SD	Control (*n* = 40) Mean ± SD	*P* value
Age, years	46.44 ± 13.97	49.16 ± 13.19	37.23 ± 15.07	< 0.001
Males, no. (%)	38 (50.7)	27 (36.0)	19 (47.5)	0.174
DM, no. (%)	25 (33.3%)	27 (36%)	13 (32.5%)	0.912
HTN, no. (%)	52 (69.3%)	49 (65.3%)	12 (30%)	< 0.001
*Laboratory tests*				
Hb, g/dL	8.50 ± 1.63	8.95 ± 1.85	11.18 ± 2.76	< 0.001
Urea, mg/dL	167.95 ± 71.22	133.42 ± 59.38	36.25 ± 17.85	< 0.001
Creatinine, mg/dL	9.09 ± 4.41	5.71 ± 2.89	0.87 ± 0.17	< 0.001
eGFR	–	12.37 ± 8.14	98.94 ± 25.53	< 0.001
K, mEq/L	4.47 ± 0.78	4.30 ± 0.71	4.23 ± 0.57	0.156
Mg, mEq/L	2.00 ± 0.37	1.95 ± 0.23	1.98 ± 0.22	0.708
Ca, mg/L	7.44 ± 1.24	7.90 ± 1.19	8.86 ± 0.60	< 0.001
PO4, mEq/L	6.13 ± 1.79	5.45 ± 1.64	3.38 ± 0.67	< 0.001
PTH, pg/mL	368.56 ± 198.75	283.17 ± 127.11	48.08 ± 16.24	< 0.001
Iron, µg/dL	56.04 ± 35.40	62.91 ± 33.20	69.52 ± 42.23	0.079
TIBC, µg/dL	195.16 ± 42.11	183.21 ± 49.32	225.02 ± 104.46	0.122
Transferrin Sat, %	29.72 ± 18.09	34.56 ± 15.21	36.28 ± 19.77	0.029
LVEF, %	66.55 ± 6.99	65.70 ± 7.63	63.75 ± 6.61	0.170

Ca, Calcium; DM, Diabetes Mellitus; eGFR, Estimated Glomerular Filtration Rate; Hb, Hemoglobin; HTN, Hypertension; K, Potassium; LVEF, Left Ventricular Ejection Fraction; Mg, Magnesium; PO4, Phosphorus; PTH, Parathyroid hormone; TIBC, Total Iron Binding Capacity

Patients with ESRD had significantly higher QTc max, QTc dispersion, and P-WD when compared to the other 2 study groups, Table 2.

**Table 2:** Electrocardiography parameters of the study population

	ESRD (*n* = 75) Mean ± SD	CKD (*n* = 75) Mean ± SD	Control (*n* = 40) Mean ± SD	*p* value
QTc max	484.59 ± 42.81	479.67 ± 41.11	452.93 ± 41.75	0.001
QTc min	397.19 ± 43.59	402.80 ± 46.84	393.68 ± 41.64	0.549
QTc average	441.90 ± 39.28	442.40 ± 40.01	424.44 ± 39.02	0.038
QTc dispersion	88.17 ± 31.89	76.93 ± 30.99	59.25 ± 22.15	< 0.001
P-WD	47.97 ± 17.68	46.32 ± 22.30	34.70 ± 13.36	0.001
Tp-e/QTc	0.64 ± 0.30	0.68 ± 0.28	0.47 ± 0.24	0.002

Max, maximum; Min, minimum; P-WD, P wave dispersion; QTc, Corrected QT interval; Tp-e, T wave peak to end interval

Comparison between patients with ESRD and patients with CKD showed non-significant differences except for levels of urea (*p* = 0.002), creatinine (*p* < 0.001), PO4 (*p* = 0.016) and PTH (*p* = 0.002) which were higher in the ESRD group, while serum calcium level (*p* = 0.023) was significantly lower in the ESRD group.

Subgroup analysis of patients with ESRD showed that males had a significantly higher P-WD values (*p* = 0.045), insignificantly higher QTc dispersion (*p* = 0.445), and insignificantly lower Tp-e/QT ratio (*p* = 0.252) when compared to females. While in the CKD group, only the Tp-e/QT ratio was significantly higher in males, Table 3.

**Table 3:** Subgroup analysis of the ECG parameters in patients with ESRD and patients with CKD

	Gender			HTN			DM		
	Male	Female	*P*	Yes	No	*P*	Yes	No	*p*
**ESRD**	n=38	n=37		n=52	n=23		n=25	n=50	
QTc dispersion	90.5 ± 34.1	85.8 ± 29.8	0.445	89.3 ± 32.0	85.7 ± 32.2	0.904	89.9 ± 31.8	87.3 ± 32.2	0.665
P-WD	51.7 ± 18.4	44.2 ± 16.3	0.045	44.5 ± 17.0	55.9 ± 17.0	0.008	51.6 ± 15.8	46.2 ± 18.4	0.188
Tp-e/QTc	0.6 ± 0.3	0.7 ± 0.3	0.252	0.7 ± 0.3	0.6 ± 0.4	0.135	0.6 ± 0.3	0.7 ± 0.3	0.196
**CKD**	n=27	n=48		n=49	n=26		n=27	n=48	
QTc dispersion	85.3 ± 30.8	72.2 ± 30.4	0.068	79.2 ± 31.9	72.7 ± 29.3	0.385	76.1 ± 32.6	77.4 ± 30.4	0.720
P-WD	51.2 ± 24.0	43.6 ± 21.0	0.272	47.8 ± 24.2	43.6 ± 18.3	0.772	48.0 ± 24.5	45.4 ± 21.2	0.740
Tp-e/QTc	0.8 ± 0.3	0.6 ± 0.3	0.043	0.7 ± 0.3	0.62 ± 0.3	0.208	0.7 ± 0.3	0.7 ± 0.3	0.699

P-WD, P wave dispersion; QTc, Corrected QT interval; Tp-e, T wave peak to end interval

ESRD hypertensive patients showed a significantly lower P-WD when compared to ESRD normotensive individuals, Table 3. On the other hand, patients with DM did not show any significant difference of the ECG parameters neither in the ESRD nor in the CKD patients as compared to non-diabetic patients.

Multivariate linear regression analysis of ESRD patients showed that serum creatinine (*β* = 0.279, *p* = 0.012) and transferrin saturation (*β* =  − 0.333, *p* = 0.003) were independent predictors of increased QTc dispersion while ejection fraction (*β* = 0.320, *p* = 0.002), hypertension (*β* =  − 0.319, *p* = 0.002), hemoglobin level (*β* =  − 0.345, *p* = 0.001), male gender (*β* =  − 0.274, *p* = 0.009) and TIBC (*β* =  − 0.220, *p* = 0.030) were independent predictors of increased P-WD. On the other hand, age (*β* =  − 0.296, *p* = 0.010) and serum creatinine (*β* =  − 0.295, *p* = 0.010) were independent predictors of Tp-e/ QT ratio, Table 4.

**Table 4:** Predictors of ECG changes in the ESRD and CKD groups

		Beta coefficient	*P*-value
ESRD			
QTc dispersion	Transferrin Saturation	− 0.333	0.003
	Creatinine	0.279	0.012
P-WD	HTN	− 0.319	0.002
	EF	0.320	0.002
	Hb	− 0.345	0.001
	Male gender	− 0.274	0.009
	TIBC	− 0.220	0.030
Tp-e/QT	Age	− 0.296	0.010
	Creatinine	− 0.295	0.010
CKD			
QTc dispersion	TIBC	− 0.285	0.013
Tp-e/QT	Ca	0.244	0.031
	Male gender	− 0.227	0.044

Ca, Calcium; EF, Ejection Fraction; Hb, Hemoglobin; HTN, Hypertension; P-WD, P wave dispersion; QTc, Corrected QT interval; TIBC, Total Iron Binding Capacity; Tp-e, T wave peak to end interval

In the CKD group, multivariate linear regression analysis showed that TIBC (*β* =  − 0.285, *p* = 0.013) was an independent predictor of QTc dispersion while serum calcium (*β* = 0.320, *p* = 0.002) and male gender (*β* =  − 0.274, *p* = 0.009) were independent predictors of Tp-e/QT ratio. However, no parameters predicted an increased P-WD in multivariate linear regression, Table 4.

The pattern of abnormal ECG changes in the study groups is shown in Table 5.

**Table 5:** The frequency of abnormal ECG changes in the study groups

	CKD (*n* = 75) No. (%)	ESRD (*n* = 75) No. (%)	Normal (*n* = 40) No. (%)
PWD ˃36 ms	40 (53.3)	50 (66.7)	13 (32.5)
QTc dispersion ˃60 ms	48 (64.0)	57 (76.0)	14 (35.0)
Tp-e/QT ˃0.25	73 (79.3)	70 (93.3)	38 (95.0)

**Fig. 1:**
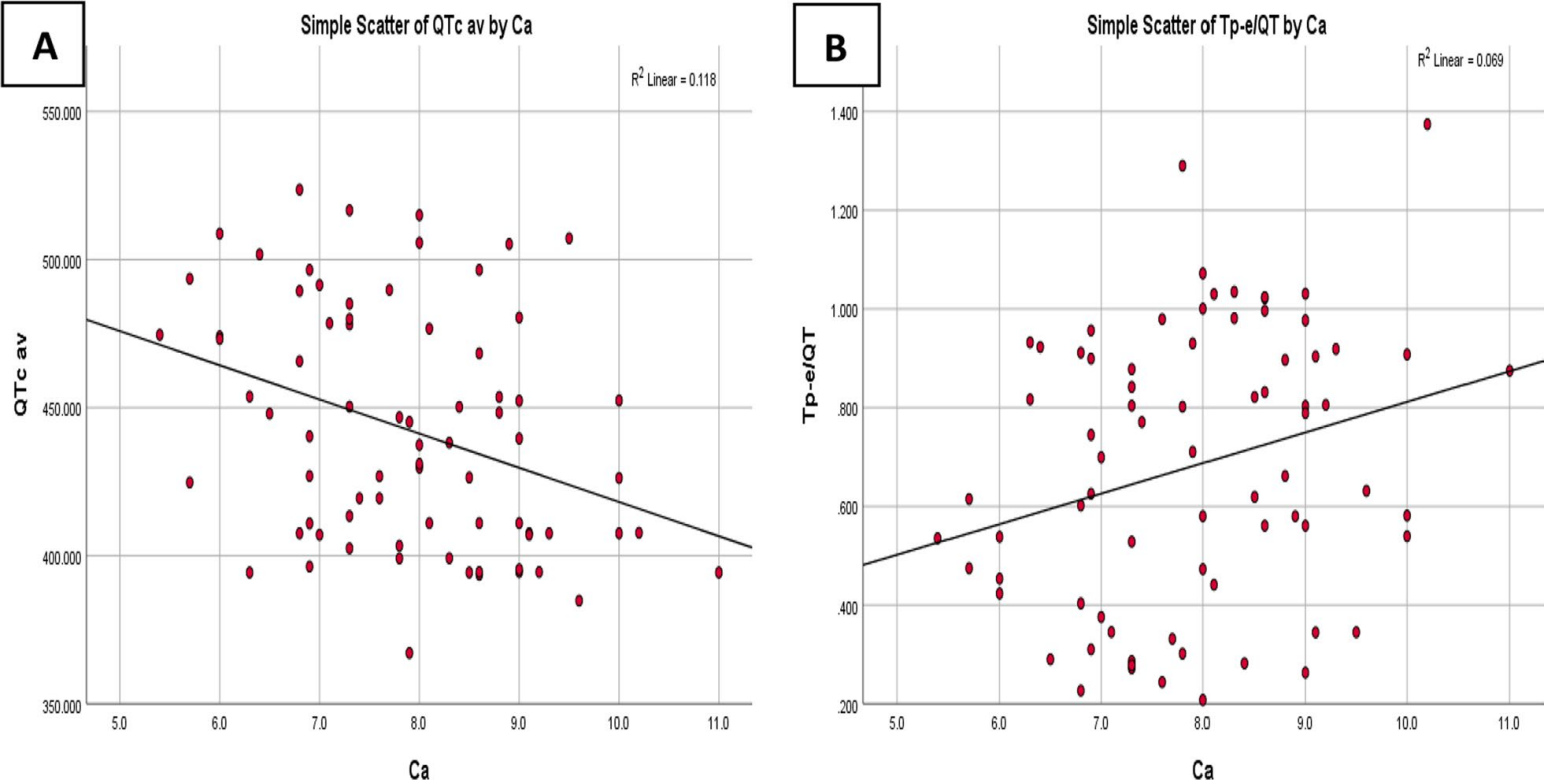
**A**: Correlation between Calcium level and QTc average in CKD patients, **B**: Correlation between Calcium level and Tp-e/QT in CKD patients

**Fig. 2:**
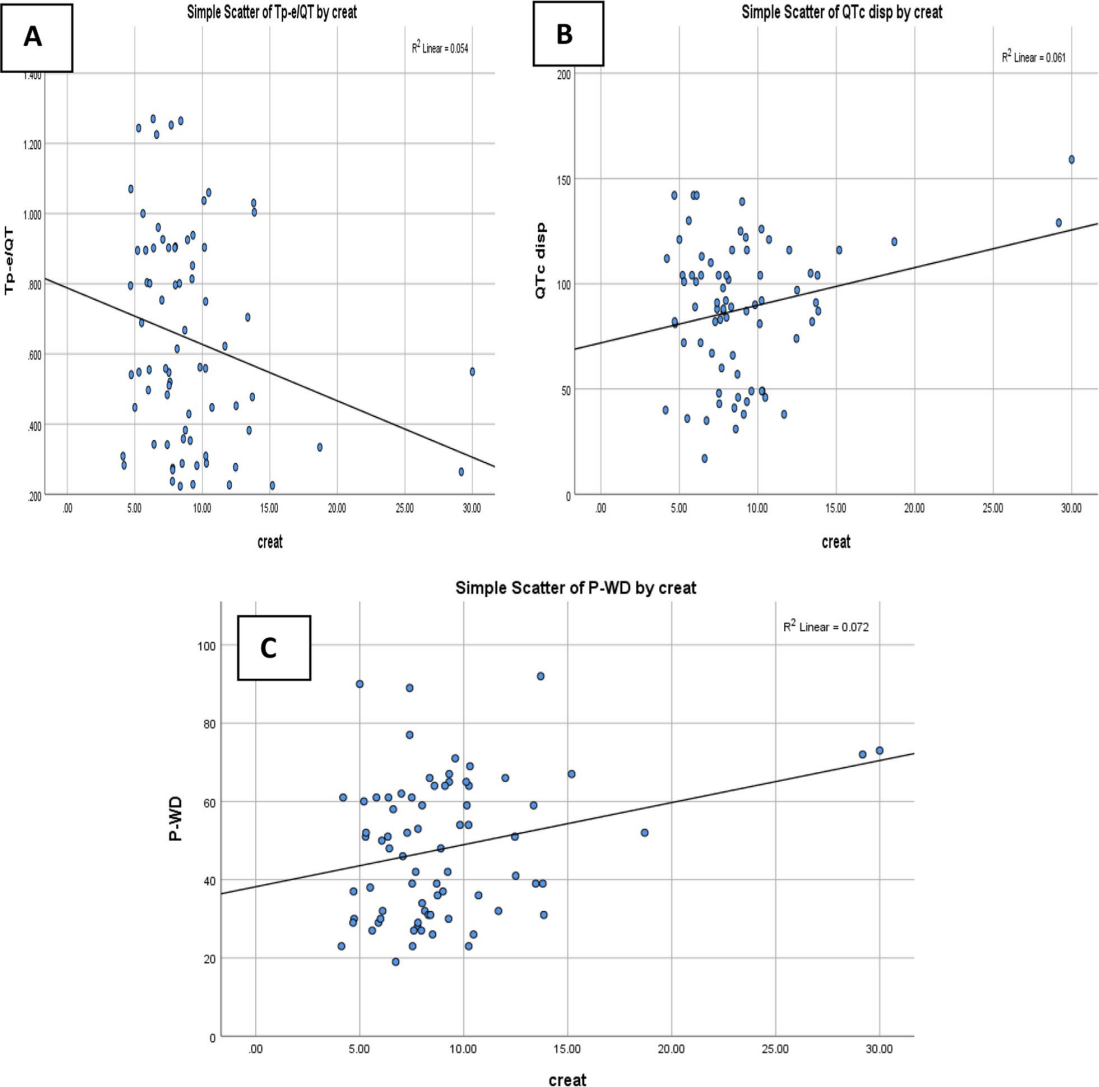
**A**: Correlation between serum creatinine level and Tp-e/QT in ESRD, **B**: Correlation between serum creatinine level and QTc in ESRD, **C**: Correlation between serum creatinine level and P-WD in ESRD

Calcium was significantly correlated to QTc average (*r* = − 0.344, *p* = 0.003) and Tp-e/QT (*r* = 0.263, *p* = 0.022), in CKD patients, Fig. 1, while creatinine level was significantly correlated to QTc dispersion (*r* = 0.247, *p* = 0.032), P-WD (*r* = 0.268, *p* = 0.020), Tp-e (*r* = − 0.239, *p* = 0.039) and Tp-e/QT (*r* = − 0.233, *p* = 0.044) in ESRD patients, Fig. 2.

## Discussion

Chronic kidney disease (CKD) is defined as either kidney damage or a decreased glomerular filtration rate (eGFR) to less than 60 mL/min/1.73 m^2^ for at least 3 months. Whatever the underlying etiology, once the loss of nephrons and reduction of functional renal mass reaches a critical point, the remaining nephrons begin a process of irreversible sclerosis that leads to a progressive decline in the GFR [12].

Patients with CKD are vulnerable to cardiac arrhythmia and in many cases, renal dysfunction stimulates arrhythmia and arrhythmia exacerbates renal dysfunction [2]. In a recent study, the ARIC study, a 2-week cardiac monitoring of patients with CKD revealed a high prevalence of non-sustained ventricular tachycardia (30.2%) and AF (7.4%), while ventricular ectopy was present in more than 90% of patients [13]. The mechanism of arrhythmia in CKD may be due to electrolyte disturbances and/or the damage caused to kidneys and heart tissues by common comorbidities like hypertension and diabetes mellitus [2]. Arrhythmia causes premature death in the general population as well as in patients with CKD [2]. Sudden Cardiac Death (SCD) is common in CKD patients and it is the most common cause of death in dialysis patients [14].

Because most arrhythmias are intermittent and usually asymptomatic, documentation and diagnosis pose a challenge to the treating physician [2]. Several electrocardiographic (ECG) methods can be used to assess the risk of arrhythmia and this includes measurement of the P-WD, QTc interval, Tp-e interval, and QTc dispersion on the standard resting 12-lead ECG. P-WD is a non-invasive ECG marker for atrial remodeling and a predictor of atrial fibrillation (AF) [15]. Increased QTc dispersion indicates heterogeneity in ventricular repolarization, which is associated with an increased risk of ventricular arrhythmia and sudden cardiac death [16] while the Tp-e interval reflects the transmural dispersion of repolarization, and a relationship was found between Tp-e and ventricular arrhythmias, heart failure, and sudden cardiac death [17]. An increased Tp-e/QT was associated with arrhythmogenesis and the underlying mechanism was functional reentry [10].

This study aims to evaluate the substrates of arrhythmia (P-WD, QTc dispersion and Tp-e/QT ratio) in ESRD patients on regular hemodialysis (no. = 75) and in patients with CKD stages 3–5 without RRT (no. = 75) all without any clinically manifest heart disease as compared to 40 normal control subjects.

Baseline data showed that ESRD patients had a higher prevalence of hypertension and their laboratory data showed lower hemoglobin, and calcium levels and higher phosphorus and parathormone levels as compared to CKD patients and control subjects.

QTc dispersion was found to be significantly higher in ESRD and CKD patients as compared to normal controls. These data are in accordance with Kolluet al., who conducted their study on 133 patients with CKD stages 3–5 without RRT and 32 healthy controls and found that QTc dispersion values were higher in patients with CKD stages 3–5 on no RRT [11].

On the other hand, Covic et al., reported that hemodialysis increases QTc interval, but not QTc dispersion in 68 stable, non-diabetic dilaysis patients with ESRD without manifest cardiac disease [18].

The reason for the increased QTc dispersion could be explained by the study performed by Jaroszyński et al., who reported that the changes of serum calcium, phosphorus, potassium and extracellular volume during hemodialysis affected QTc dispersion and could promote ventricular arrhythmogenesis [19].

Tp-e/QT ratio was significantly higher in ESRD and CKD patients when compared to normal controls. These results are similar to the results found by Kollu et al., who found that Tp-e/QT is increased in CKD stages 3–5 patients without RRT [11] and Guclu et al. who found that Tp-e, Tp-e/QT, Tp-e/QT, and QTc were significantly higher in the hemodialysis and peritoneal dialysis patients [20]. The mechanism of ventricular repolarization prolongation in patients with ESRD was associated with traditional and CKD-related risk factors. Several CKD-related risk factors such as inflammation [21], hyperferritinemia, hyperparathyroidism, hyperphosphatemia [22], and structural changes in uremic myocardium are frequently present in patients with CKD. An increased prevalence of left ventricular dysfunction, autonomic dysfunction, myocyte dysfunction, altered electrolyte metabolism, and cardiac fibrosis may also contribute to arrhythmic risk in patients with kidney disease [23]. On the contrary, Karaagac et al. found that Tp-e/QT values were similar in ESRD patients receiving hemodialysis compared to healthy age and gender matched individuals [24].

P-WD was significantly higher in ESRD and CKD patients as compared to normal controls. This finding is similar to what was found by Kollu et al. who reported increased P-WD in CKD stages 3–5 patients without RRT [11]. Drighil et al. studied the impact of dilaysis on P-WD in 17 patients undergoing hemodialysis (HD). They found that P-WD decreased, and left atrial dimension decreased, after HD. The change in P-WD was correlated with fluid removed by HD and the subsequent decrease in LA dimensions [25].

Intracellular or intercellular factors may lead to site‐specific conduction differences [26]. P-WD increases with age and in patients with structural heart disease. Hypertension, coronary heart disease, valvular heart disease, pericarditis, dilated cardiomyopathy, ionic disturbances, and autonomic dysfunction are common underlying diseases associated with increased P-WD. Most patients with CKD have these abnormalities [27].

In the comparison of the ESRD group to the CKD group, we could not find a significant difference regarding prolonged QTc dispersion, prolonged P-WD duration or increased Tp-e/QT ratio.

P-WD values were significantly higher in males in the ESRD group and Tp-e/QT ratios were significantly higher in males in the CKD group. The studies which evaluated P-WD and Tp-e/QT in renal patients did not compare sex-related differences. Women are known to have a longer QTc interval, and a lower QTc dispersion than men. The mechanism for the longer QTc interval in women is not completely known but it does not appear to be related to the acute effects of estrogen or progesterone or differences in autonomic innervation [28].

Despite the fact that DM increases the risk of ventricular arrhythmogenesis [29], QTc dispersion, P-WD and Tp-e/QT in diabetic patients with ESRD and CKD were comparable to non-diabetic patients. This is consistent with Kollu et al. who found no difference between diabetic and non-diabetic population in terms of the duration of P-WD, QTc and Tp-e interval and also Tp-e/QT ratio [11]. On the contrary, a study published by Tokatli et al. reported that the values of Tp-e, Tp-e/QT ratio were higher in patients with type 2 DM [30].

On multivariate linear regression analysis in the ESRD group, transferrin saturation was the only independent predictor of an increased QTc dispersion while hemoglobin and male gender were the independent predictors of an increased P-WD. In the CKD group, the serum calcium and male gender were the independent predictors of an increased TP-e/ QT ratio.

## Conclusions

Patients with stage 3–5 chronic kidney disease and those with end-stage renal disease on regular hemodialysis exhibit significant ECG changes that could be substrates for ventricular and supraventricular arrhythmias. Those changes were more evident in patients on hemodialysis. Larger and more comprehensive studies are required for the assessment of ECG changes evolution from chronic kidney disease to hemodialysis and to evaluate the value of these ECG abnormalities in predicting cardiac arrhythmias.

## Data Availability

The datasets used and/or analyzed during the current study are available from the corresponding author on reasonable request.

## Abbreviations

CKD: Chronic kidney disease
CVD: Cardiovascular diseases
DM: Diabetes mellitus
ECG: Electrocardiogram
eGFR: Estimated Glomerular filtration rate
ESRD: End-stage renal disease
HD: Hemodialysis
HTN: Hypertension
P-WD: P wave dispersion
QTc: Corrected QT interval
RRT: Renal replacement therapy
SCD: Sudden Cardiac Death
TIBC: Total iron binding capacity
Tp-e: T wave peak to end interval

## Availability of data and materials

The datasets used and/or analyzed during the current study are available from the corresponding author on reasonable request.
